# Early Warning Scores to Support Continuous Wireless Vital Sign Monitoring for Complication Prediction in Patients on Surgical Wards: Retrospective Observational Study

**DOI:** 10.2196/44483

**Published:** 2023-08-30

**Authors:** Mathilde C van Rossum, Robin E M Bekhuis, Ying Wang, Johannes H Hegeman, Ellis C Folbert, Miriam M R Vollenbroek-Hutten, Cornelis J Kalkman, Ewout A Kouwenhoven, Hermie J Hermens

**Affiliations:** 1 Department of Biomedical Signals and Systems University of Twente Enschede Netherlands; 2 Department of Cardiovascular and Respiratory Physiology University of Twente Enschede Netherlands; 3 Department of Surgery Hospital Group Twente Almelo Netherlands; 4 Hospital Group Twente Academy Hospital Group Twente Almelo Netherlands; 5 Department of Anesthesiology University Medical Center Utrecht Utrecht Netherlands

**Keywords:** early warning scores, vital signs, telemedicine, physiological monitoring, clinical alarms, postoperative complications, perioperative nursing

## Abstract

**Background:**

Wireless vital sign sensors are increasingly being used to monitor patients on surgical wards. Although early warning scores (EWSs) are the current standard for the identification of patient deterioration in a ward setting, their usefulness for continuous monitoring is unknown.

**Objective:**

This study aimed to explore the usability and predictive value of high-rate EWSs obtained from continuous vital sign recordings for early identification of postoperative complications and compares the performance of a sensor-based EWS alarm system with manual intermittent EWS measurements and threshold alarms applied to individual vital sign recordings (single-parameter alarms).

**Methods:**

Continuous vital sign measurements (heart rate, respiratory rate, blood oxygen saturation, and axillary temperature) collected with wireless sensors in patients on surgical wards were used for retrospective simulation of EWSs (sensor EWSs) for different time windows (1-240 min), adopting criteria similar to EWSs based on manual vital signs measurements (nurse EWSs). Hourly sensor EWS measurements were compared between patients with (event group: 14/46, 30%) and without (control group: 32/46, 70%) postoperative complications. In addition, alarms were simulated for the sensor EWSs using a range of alarm thresholds (1-9) and compared with alarms based on nurse EWSs and single-parameter alarms. Alarm performance was evaluated using the sensitivity to predict complications within 24 hours, daily alarm rate, and false discovery rate (FDR).

**Results:**

The hourly sensor EWSs of the event group (median 3.4, IQR 3.1-4.1) was significantly higher (*P*<.004) compared with the control group (median 2.8, IQR 2.4-3.2). The alarm sensitivity of the hourly sensor EWSs was the highest (80%-67%) for thresholds of 3 to 5, which was associated with alarm rates of 2 (FDR=85%) to 1.2 (FDR=83%) alarms per patient per day respectively. The sensitivity of sensor EWS–based alarms was higher than that of nurse EWS–based alarms (maximum=40%) but lower than that of single-parameter alarms (87%) for all thresholds. In contrast, the (false) alarm rates of sensor EWS–based alarms were higher than that of nurse EWS–based alarms (maximum=0.6 alarm/patient/d; FDR=80%) but lower than that of single-parameter alarms (2 alarms/patient/d; FDR=84%) for most thresholds. Alarm rates for sensor EWSs increased for shorter time windows, reaching 70 alarms per patient per day when calculated every minute.

**Conclusions:**

EWSs obtained using wireless vital sign sensors may contribute to the early recognition of postoperative complications in a ward setting, with higher alarm sensitivity compared with manual EWS measurements. Although hourly sensor EWSs provide fewer alarms compared with single-parameter alarms, high false alarm rates can be expected when calculated over shorter time spans. Further studies are recommended to optimize care escalation criteria for continuous monitoring of vital signs in a ward setting and to evaluate the effects on patient outcomes.

## Introduction

### Background

Surgical patients are at risk of developing postoperative complications, which may progress to life-threatening illnesses and seriously affect patient outcomes if not promptly detected and correctly treated [1]. Most postoperative complications occur in the first week after surgery and are typically present in a ward setting [2-5]. Therefore, adequate patient monitoring in surgical wards is crucial for identifying the early signs of complications [6].

In hospital wards, patient monitoring typically consists of routine vital sign checks performed by nurses every 6 to 8 hours, complemented by subjective evaluation of the patient status during nursing activities [7,8]. In addition, early warning scores (EWSs) are widely used to evaluate the risk of patient deterioration. EWSs are typically calculated by assigning points to a measured set of vital signs, where the sum of the points reflects the EWSs. EWSs are often implemented as part of rapid response systems, where they are used to trigger clinical actions or escalation of care when exceeding a predefined threshold [5,9]. However, vital sign checks and corresponding EWSs are often incomplete or not performed on time, particularly during the night or when the protocol mandates more frequent measurements in high-risk patients [10,11]. Together with the intermittent measurement frequency, this may lead to unnoticed or delayed detection of patient deterioration.

In recent years, wireless sensors that enable mobile vital sign monitoring have been introduced. These sensors facilitate automated, less obtrusive, and continuous patient monitoring in a ward setting [12,13]. Although there is still little evidence regarding the clinical effects of continuous monitoring in this setting, various studies have suggested that continuous monitoring can aid early identification of clinical deterioration and may provide opportunities to improve outcomes in patients with complications [4,14]. However, the interpretation of the large amount of data that are generated by the sensors is still a major challenge because vital sign measurements fluctuate largely during the day and are influenced by movement and many patient-related or environmental factors [15]. Moreover, continuous manual data observation is hampered by restricted staffing levels in a ward setting and inconsistent assessment of abnormalities by caregivers [16]. Therefore, to promote an adequate and timely response to patient deterioration, automatic methods to support and identify vital sign abnormalities related to potential complications are desired.

Currently, wireless monitoring is often implemented in combination with traditional alarm strategies, where an alert is sent automatically as soon as the measurement of one of the vital signs exceeds a preset upper or lower threshold [12,17]. Although this single-parameter alarm strategy is standard in high-care units such as intensive care units, these alarms are sensitive to various disturbances and easily lead to excessive false alarm rates. As the nurse:patient ratio is lower in a ward setting, this alarm burden is a serious concern for nurse workload and could lead to alarm fatigue, thereby potentially threatening patient safety [7]. Furthermore, single-parameter abnormality detection does not align with the current use of EWSs for risk prediction in patients on surgical wards, which relies on multiple parameters.

### Objective

Accordingly, it may be of interest to use EWSs instead of single-parameter alarms to detect potential abnormalities in continuously monitored patients on surgical wards, as supported by recently developed remote monitoring systems [12]. However, there is still little evidence regarding whether EWSs derived from mobile vital sign measurements can actually support the identification of deterioration in patients on surgical wards and be useful as alarm systems for continuous remote monitoring. Therefore, this exploratory study aimed to gain insight into the potential sensitivity of a sensor-based, high-rate EWS for predicting postoperative complications and to evaluate the expected daily alarm rate in comparison with single-parameter alarm criteria as well as manual EWS measurements for different alarm settings. Furthermore, the potential predictive value of “nurse worry” and patient-reported deterioration was explored to investigate the possible benefits of systematically collecting subjective data in monitoring routines.

## Methods

### Study Design

The study had an observational retrospective design.

### Ethics Approval

This study was approved by The Medical Research Ethics Committee Twente (MoViSign study; NL65885.044.18).

### Informed Consent

All included participants provided written informed consent to participate in the study and to use their data for research purposes.

### Population

The study included patients (aged ≥18 years) undergoing elective esophageal or gastric resection (ie, upper gastrointestinal [GI] group) and older patients (aged ≥70 years) undergoing acute surgery for a hip fracture (ie, hip fracture group), as these groups are known to have relatively high rates of complications during ward stay. Patients were recruited preoperatively or after postoperative admission to the surgical ward (GI unit or center of geriatric traumatology). Patients were excluded if they had implanted electronic medical devices, had known allergies to materials used in wearable sensors, had suspected delirium or cognitive impairment, or were unable to decide upon study participation. Recruitment was performed during office hours only, and patients who were expected to leave the hospital within 24 hours after possible recruitment were not eligible for study participation.

### Measurements

The patients received standard ward care including routine nursing observations. Nurses were instructed to perform manual measurements of vital signs and calculate a corresponding Modified Early Warning Score (MEWS) [18] at least 3 times a day. The MEWS was used to indicate the risk of patient deterioration (≤2=low risk, 3-4=intermediate risk, and ≥5=high risk), where cutoff values of 3 and 5 were used as response triggers to call a physician and the rapid response team for further patient investigation, respectively. In addition to routine care, vital signs were recorded every minute using a wearable patient monitoring system. Heart rate (HR) and respiratory rate (RR) were measured using the chest-worn LifeTouch sensor (Isansys Lifecare Ltd). Axillary temperature (AT) was recorded with the LifeTemp sensor (Isansys Lifecare Ltd), and blood oxygen saturation (SpO_2_) was measured with the 3150 WristOx finger pulse oximeter (Nonin Medical Inc). All the measurements were sent to a bedside patient gateway using an encrypted Bluetooth connection. Sensor recordings were started after the patient was admitted to the surgical ward and informed consent was obtained and continued until hospital discharge (or premature patient dropout). During the recording period, the researchers checked and maintained the technical functionality of the sensor recordings at least once a day on weekdays. Medical professionals and patients were blinded to the sensor measurements to prevent bias, but nurses were instructed to temporarily detach the sensors for showering, diagnostic imaging, or surgical interventions or reinterventions.

To explore the added value of routine collection of subjective information from nurses and patients, nurses were instructed to fill in a paper checklist during every nurse shift to register the possible presence and reasons for nurse worry using an adapted version of the Dutch Early Nurse Worry Indicator Score, as specified in Multimedia Appendix 1 [19]. Furthermore, patients were asked to fill in a daily diary during their ward stay to indicate the presence of symptoms, how they were currently feeling (0 points=very poor; 5 points=very good), and how they felt compared with the previous day (0 points=much impaired; 5 points=much improved) on a 5-point Likert scale. If needed, researchers helped or encouraged the patients to fill in the diary when they visited the patients. The nurse worry checklist and patient diary were used only for research purposes. All collected measurements were deidentified for further analysis.

### Events

An event was defined as a postoperative complication that was diagnosed according to local standards and that was treated during ward stay or within 7 days after hospital discharge. Events were identified retrospectively from patient records and reviewed by a surgeon (EAK or HJH) to ensure correct interpretation. As there is no way to ascertain exactly when a complication started, we recorded the start of targeted treatment as the onset time of the complication. Similarly, the end time of the complication was defined as the timing of the last therapeutic action, for example, the last medication gift. If no information regarding the last therapeutic action was available or if therapy was continued after hospital discharge (or premature patient dropout) or in case of palliative treatment, it was assumed that the complication lasted until the end of the sensor recording period. Complications classified as Clavien-Dindo class [20] of II or higher, for which treatment was started within the measurement period (ie, the period of sensor measurements during ward stay), were eligible and included for further analysis. In patients with multiple complications, eligible events were only included if treatment was started at least 24 hours after any previously included complication. Furthermore, complications were only included if at least 4 hours of HR and RR sensor measurements were available in the 24 hours window before the onset time of the event after data preprocessing (see the *Data Preprocessing* section). Patients with eligible complications were enrolled in the event group, whereas patients with an uncomplicated postoperative trajectory served as the control group. Patients with only ineligible complications were excluded from further analyses. To evaluate the risk of selection bias, the baseline characteristics were compared between the included and excluded patient groups.

### EWS and Alarm Simulation

#### Overview

**Figure 1:**
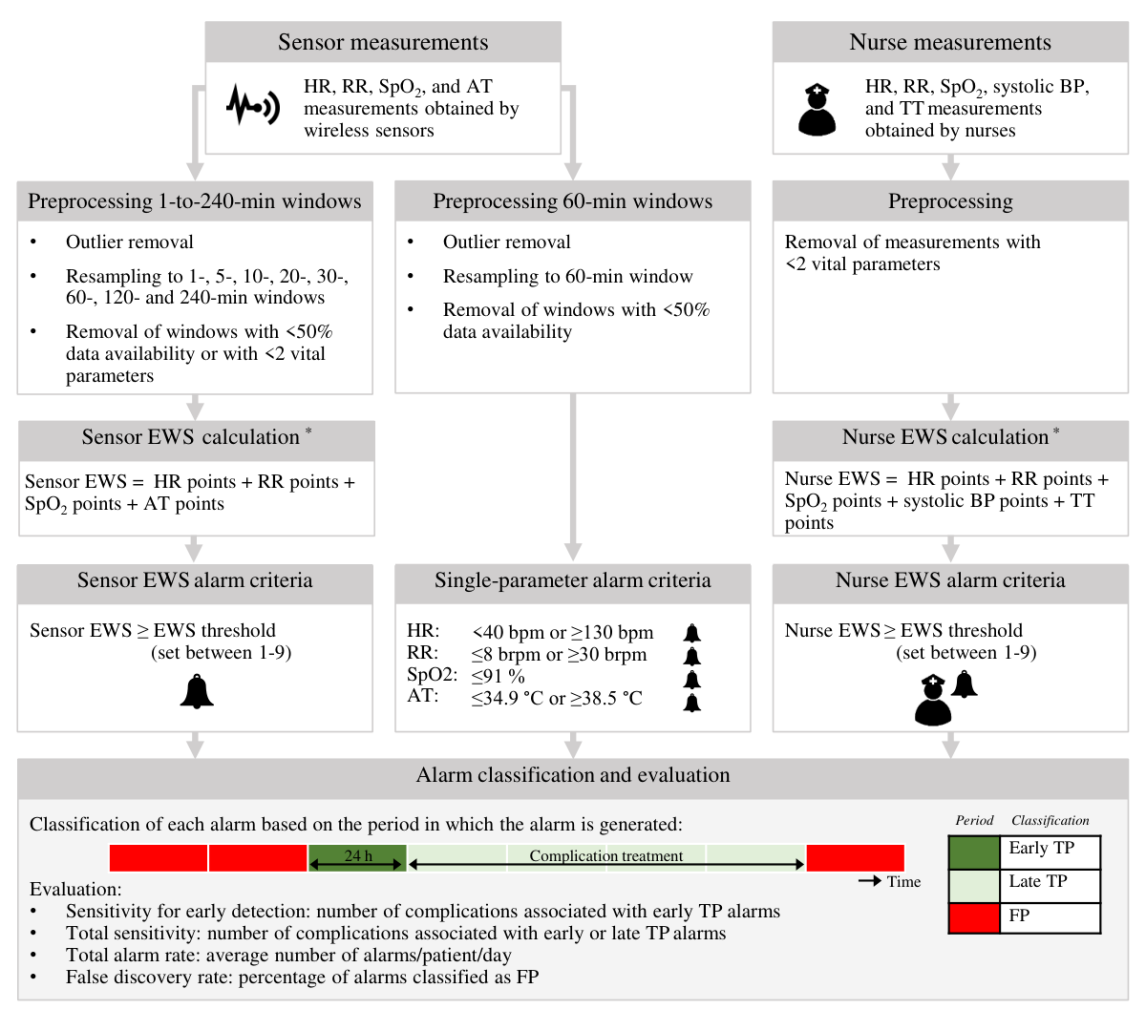
Overview of the calculation of the early warning score (EWS), alarm simulation, and alarm evaluation in sensor and nurse measurements. AT: axillary temperature; BP: blood pressure; bpm: beats per minute; brpm: breaths per minute; FP: false-positive; HR: heart rate; nurse EWS: early warning score based on manual vital signs measurements; RR: respiratory rate; sensor EWS: early warning score based on sensor vital signs measurements; SpO_2_: blood oxygen saturation; TP: true-positive; TT: tympanic temperature. *EWS calculation criteria are described in Table 1.

Sensor and nurse measurements were used to simulate corresponding EWSs. In addition, alarms were respectively simulated using the EWSs based on sensor vital signs measurements (sensor EWSs), EWSs based on manual vital signs measurements (nurse EWSs), and single vital sign recordings, with the aim of evaluating and comparing the effects of using these measurements as response triggers or active alarms. Figure 1 provides an overview of the alarm simulation and evaluation, which are further explained in the following subsections.

**Table 1:** Criteria used to calculate early warning scores (EWSs) based on sensor vital signs measurements (sensor EWSs) and early warning scores based on manual vital signs measurements (nurse EWSs).

Variable	Inclusion of vital sign			Points						
	Sensor EWSs	Nurse EWSs	3		2	1	0	1	2	3
RR^a^ (brpm^b^)	✓^c^	✓			≤8		9-14	15-20	21-29	≥30
HR^d^ (bpm^e^)	✓	✓			≤40	41-50	51-100	101-110	111-129	≥130
SpO_2_^f^ (%)	✓	✓^g^	≤91		92-93	94-95	≥96			
Systolic BP^h^ (mm Hg)		✓	≤70		71-80	81-100	101-200		≥201	
AT^i^ or TT^j^ (°C)^k^	✓	✓			≤34.9		35-38.4		≥38.5	

^a^RR: respiratory rate.

^b^brpm: breaths per minute.

^c^Variables included in the sensor EWSs and nurse EWSs.

^d^HR: heart rate.

^e^bpm: beats per minute.

^f^SpO_2_: blood oxygen saturation.

^g^No standard protocol; therefore, only included if available.

^h^BP: blood pressure.

^i^AT: axillary temperature.

^j^TT: tympanic temperature.

^k^AT used for sensor EWSs and TT used for nurse EWSs.

#### Data Preprocessing

Data preprocessing and analysis were performed using MATLAB (version R2021b; The Math Works Inc). Sensor recordings were preprocessed by removing implausible extreme values (HR >200 or <30 beats/min [bpm], RR <5 or >50 breaths/min [brpm], AT <30 °C or >50 °C, and SpO_2_ <70% or >100%), most likely caused by artifacts. As no standard monitoring frequency has currently been established for monitoring patients on surgical wards, sensor measurements were analyzed repeatedly for different time windows. Accordingly, the minute-sampled vital sign recordings were resampled by averaging the signal values in successive windows of 5, 10, 20, 30, 60, 120, and 240 minutes. For each vital parameter, windows wherein data were missing for >50% of the time were disregarded.

#### EWS Simulation

The original and down-sampled sensor measurements as well as all vital sign measurements obtained by nurses during the sensor recording period were used for the retrospective simulation of the corresponding EWSs. For this purpose, points were assigned to each vital parameter according to the MEWS criteria [18] (Table 1). As SpO_2_ is not included in the MEWS, the SpO_2_ criteria were obtained from the UK National Early Warning Score criteria [21].

The sensor EWSs were calculated as the sum of the points assigned to each of the vital parameters measured by the sensor system, resulting in sensor EWSs between 0 and 11. Similarly, the vital sign measurements obtained by nurses were used to calculate the nurse EWSs, ranging between 0 and 14. The sensor EWSs and nurse EWSs were only calculated for measurements in which at least 2 vital sign values were available.

#### Alarm Simulation

To evaluate and compare the effect of using the sensor or nurse measurements as an active notification system, “alarms” were simulated based on the sensor EWS measurements (“sensor EWS–based alarms”) and based on all available nurse EWS measurements (“nurse EWS–based alarms”). An alarm was defined as an occurrence where the EWS increased to a higher level compared with a previous measurement, thereby exceeding a preset threshold (“EWS threshold”). When the measurements exceeded the EWS threshold from the beginning of the measurement period, the first sample was considered as the first alarm. EWS alarms were simulated repeatedly for EWS thresholds of 1 to 9, aiming to evaluate the alarms for a wide range of thresholds. However, as MEWS cutoff values of 3 and 5 were used in this study’s hospital as response triggers, these thresholds were selected as EWS threshold in the primary analysis. For the sensor EWSs, the primary investigation was based on 1-hour time windows, but alarms were also simulated for all other time windows (1-240 min).

In addition to the alarms based on EWSs, sensor measurements were used to simulate single-parameter alarms, similar to traditional physiological alarm systems. A single-parameter alarm was simulated when the vital sign measurement exceeded the predefined normal range. Upper and lower thresholds were defined by the outer limits of the EWS criteria, that is, HR ≤40 or ≥130 bpm, RR ≤8 or ≥30 brpm, SpO_2_ ≤91%, and AT ≤34.9 °C or ≥38.5 °C. Single-parameter alarms were simulated for 1-hour time windows, and situations where multiple single-parameter alarms were present in the same window were counted as 1 alarm.

#### Alarm Classification

All the simulated alarms were classified based on the timing of the alarms, as illustrated in Figure 1. Alarms that were simulated ≤24 hours before the onset of the included events were classified as “early true-positive” (TP) alarms, thereby reflecting alarms that could theoretically promote early identification of events. Alarms that presented during the treatment period of the included events were classified as “late true-positive” alarms. Alarms that presented >24 hours before onset or after the end of the treatment period of included events or that presented in the control group were classified as false-positive (FP) alarms, that is, false alarms.

### Analysis

#### Availability and Agreement of Vital Signs Measurements

The availability of sensor measurements, that is, data completeness, was calculated as the percentage of the total recording time where vital sign measurements were available after preprocessing in the total study population. The availability of nurse measurements was verified by the number of measurements available per 24 hours. The agreement between nurse and sensor measurements was explored for each vital parameter using Bland-Altman analysis for repeated measures [22] by comparing each available nurse measurement with the corresponding average value of the available preprocessed sensor measurements in the 5-minute window [23] before the nurse measurement.

#### Group Comparison

All the measurements (sensor recordings, sensor EWSs, nurse EWSs, nurse worry checklist, and patient diary) were compared between the event and control groups to explore their relationships with the development of complications. For all available hourly sensor EWS and nurse EWS measurements obtained during the sensor recording period, the average EWSs and the percentage of measurements where the EWSs were ≥3 (intermediate to high risk) and ≥5 (high risk) was investigated for each group. For the sensor EWSs, the number of EWS points assigned to the hourly sensor measurement windows was evaluated to investigate the contribution of the different vital parameters to the sensor EWSs. Furthermore, the percentage of hourly sensor measurements, that is, single-parameter recordings, that exceeded the upper or lower thresholds defined for the single-parameter alarms were assessed. Finally, the prevalence of worry expressed by nurses and the prevalence of (very) poor current status and (severely) impaired status indicated by patients during the study period were evaluated to explore the potential diagnostic value of this subjective information. All group differences were statistically compared using the Mann-Whitney *U* test. In addition, a sensitivity analysis was performed to verify the impact of patient exclusion on group differences by separately evaluating group differences after adding excluded patients to the control group.

#### Alarm Evaluation

The performance of the sensor EWS–based, nurse EWS–based, and single-parameter alarms was investigated using 2 sensitivity rates: the total alarm rate (TAR) and the false discovery rate (FDR) [24]. The sensitivity for early detection of adverse events was calculated as the percentage of events associated with early TP alarms. Similarly, the total sensitivity for event detection was defined as the percentage of events for which early TP and late TP alarms were observed. The TAR was defined as the average daily number of alarms per patient and the FDR as the percentage of alarms classified as FP. The sensitivity for early detection, total sensitivity, TAR, and FDR were compared between the sensor EWS–based, nurse EWS–based, and single-parameter alarms.

## Results

### Population

A total of 60 patients were included in the study, of whom 33 (55%) were patients undergoing elective esophageal or gastric resection and 27 (45%) were older patients with a hip fracture. The baseline characteristics of the participants are presented in Table 2. On average, sensor recordings were started 48 hours after the onset of surgery. Out of 60 patients, the period of vital sign recording was completed by 46 (77%) patients, whereas measurements were stopped before hospital discharge in 14 (23%) patients due to withdrawal from study participation (9/14, 64%), patient transfer (2/14, 14%), organizational reasons (2/14, 14%), and transition to palliative care (1/14, 7%). No temperature sensor was placed in 2 patients, and pulse oximeter recordings were not started in 2 other patients due to unavailable sensors or patient refusal.

**Table 2:** Baseline characteristics of the study population.

Characteristic			Upper GI^a^ group (n=33)		Hip fracture group (n=27)
Age (years), mean (SD)			64 (11)		82 (7)
**Sex, n (%)**					
	Male	24 (73)		4 (15)	
	Female	9 (27)		23 (85)	
**American Society of Anesthesiologists classification, n (%)**					
	II	20 (61)		12 (44)	
	III	12 (36)		11 (41)	
	IV	1 (3)		3 (11)	
	Unknown	0 (0)		1 (4)	
**Comorbidities, n (%)**					
	Cardiovascular	19 (58)		19 (70)	
	Diabetes	3 (9)		7 (26)	
	GI	12 (36)		8 (30)	
	Neuropsychiatric	6 (18)		10 (37)	
	Pulmonary	6 (18)		8 (30)	
	Urogenital	9 (27)		11 (41)	
	Other categories^b^	6 (18)		11 (41)	
ICU^c^ readmission, n (%)			3 (9)		0 (0)
In-hospital mortality, n (%)			0 (0)		0 (0)
Out-of-hospital mortality^d^, n (%)			0 (0)		1 (4)
Hospital readmission^d^, n (%)			0 (0)		0 (0)

^a^GI: gastrointestinal.

^b^Includes endocrine, infective, neuromuscular, or thrombosis-related comorbidities.

^c^ICU: intensive care unit.

^d^Within 7 days of hospital discharge.

### Events

During the measurement period or the 7-day follow-up period, a total of 40 complications were observed in 28 patients. Of all observed complications (n=40), 25 (63%) events were excluded (Clavien-Dindo class I: 10/25, 40%; within 24 hours from the previous event: 2/25, 8%; no or insufficient sensor data availability: 13/25, 52%), resulting in the exclusion of 14 patients from the analysis. Out of 40 events, 15 (38%) were included in the analysis (Clavien-Dindo class II: 10/15, 67%; III: 2/15, 13%; IV: 1/15, 7%; V: 1/15, 7%). The included events were present in a group of 14 patients (upper GI group: n=9, 64%; hip fracture group: n=5, 36%), who were selected as the event group. The control group consisted of 32 patients (upper GI group: n=17, 53%; hip fracture group, n=15, 47%) in whom no events were observed. We found no statistical differences in the baseline characteristics between the event and control groups or between the included and excluded patient groups.

### Availability and Agreement of Vital Signs Measurements

Sensor recordings in the total study population had a median duration of 120 (IQR 93-163) hours. After preprocessing, the median data availability of HR and RR was 84% (IQR 74%-94%) of the measurement time and that of AT was 97% (IQR 84%-100%). SpO_2_ values were missing more frequently, with a median availability of 46% (IQR 40%-60%). The median number of vital sign observations registered by nurses was 3 (IQR 2-4) per day. On average, HR was available in 96%, RR in 48%, SpO_2_ in 92%, systolic blood pressure in 95%, and tympanic temperature in 93% of nurse observations.

Bland-Altman plots for all available nurse and sensor data pairs are presented in Multimedia Appendix 2. The sensor measurements of HR and RR were higher than the nurse measurements, with a mean difference of 3 bpm and 9.4 brpm, respectively, where the largest differences between measurements were particularly seen for higher measurement values. In contrast, the SpO_2_ (mean absolute difference 2.1%) and temperature measurements (mean difference 0.9 °C) recorded by the sensor were lower than the nurse measurements, where the largest deviations were observed in the lower ranges. For all parameters, the limits of agreement were relatively wide compared with the observed range of values.

### Group Comparison

**Figure 2:**
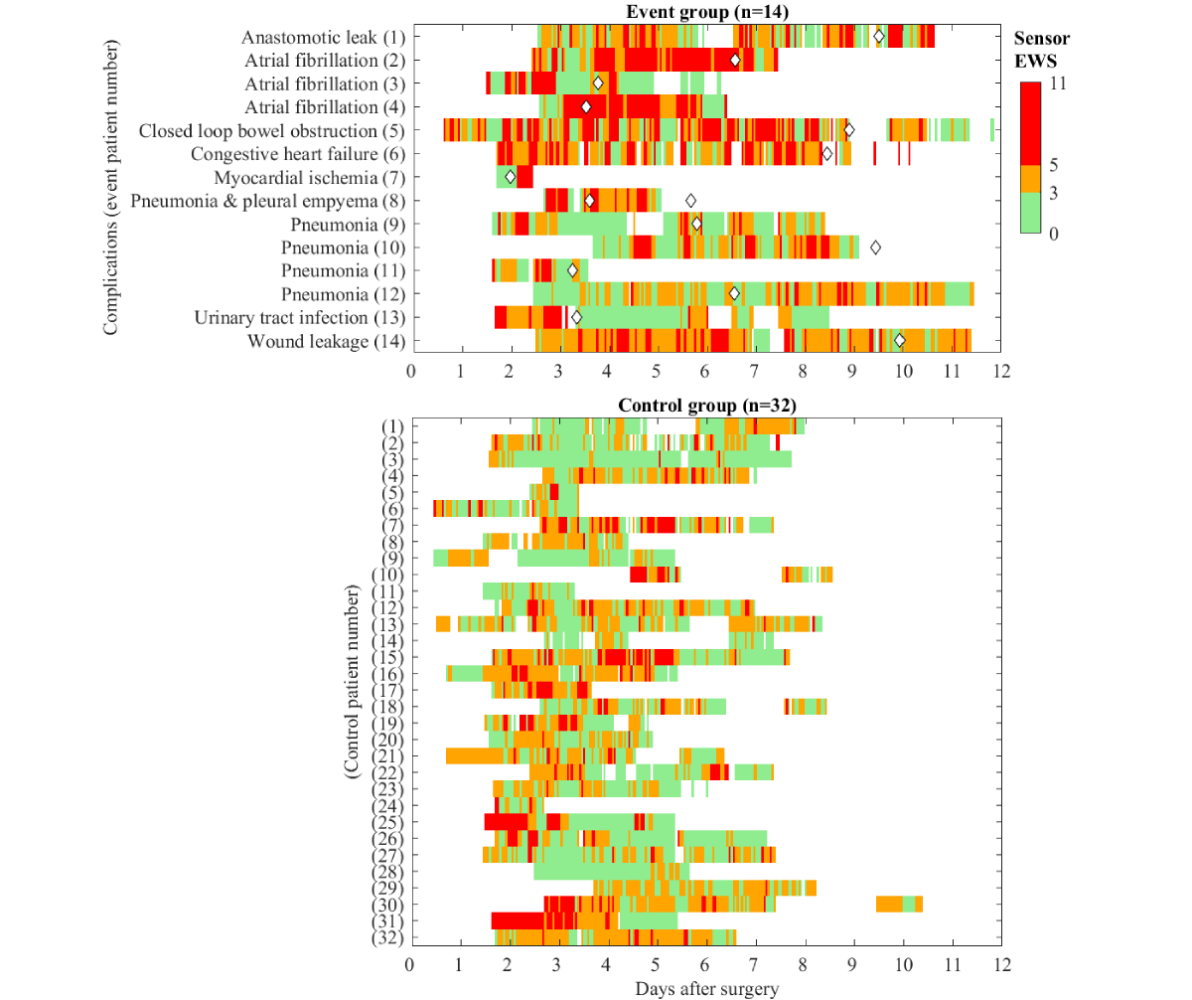
Early warning score based on sensor vital signs measurements (sensor EWS) of all patients enrolled in the event and control group, calculated using 1-hour windows and presented according to cutoff values of ≥3 (orange) and ≥5 (red). Complication timing (onset of treatment) is annotated by white diamond markers.

**Figure 3:**
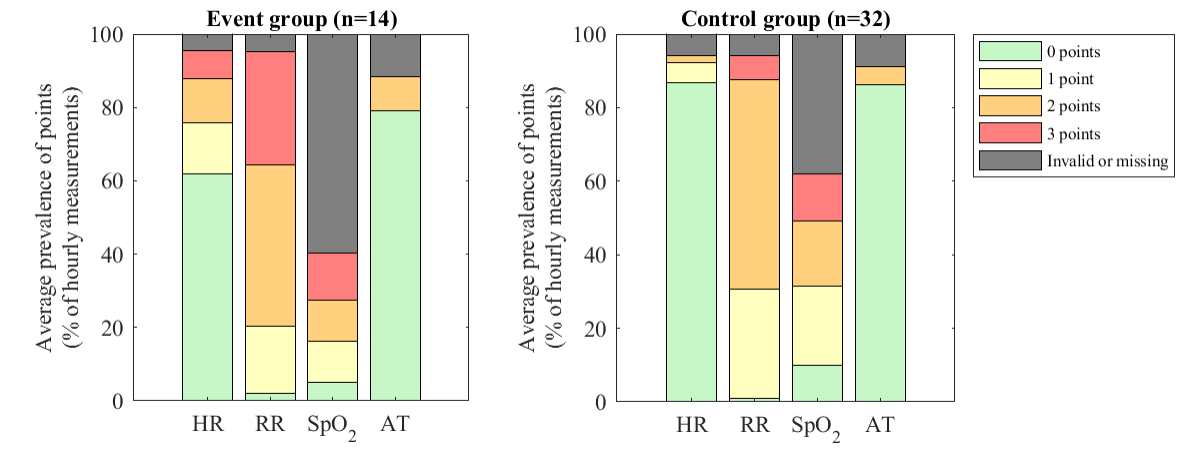
Number of points that were assigned to the hourly sensor vital sign measurements in patients with and without postoperative complications following the criteria used to calculate early warning scores (specified in Table 1). The bar stacks reflect the average percentage of 1-hour windows in which 0, 1, 2, or 3 points were assigned to the corresponding vital parameter. AT: axillary temperature; HR: heart rate; RR: respiratory rate; SpO_2_: blood oxygen saturation.

Figure 2 shows the hourly sensor EWSs over time for the event and control groups, visualized using EWS thresholds of 3 and 5. High sensor EWSs were often scattered over time, and no uniform sensor EWS pattern was observed in the days or hours preceding the complication treatment. According to the average number of points that were assigned to the hourly vital sign measurements (Figure 3), RR and SpO_2_ received 2 or 3 points in at least half of the available measurements for both the event and control groups, thereby contributing most to the sensor EWSs.

Table 3 describes the differences in the measurements between the event and control groups. The average hourly sensor EWSs of the included patients with complications were significantly higher (*P*=.004) than those of the patients with uncomplicated postoperative trajectories. In addition, the sensor EWSs in the event group reached scores of ≥5 more often compared with the control group (*P*<.001). For nurse EWSs, the number of measurements, as well as the average nurse EWSs and the percentage of observations where sensor EWSs of ≥3 or ≥5, were significantly higher for the event group (*P*=.02, *P*=.009, *P*<.001, and *P*<.001, respectively). Furthermore, nurses expressed worry more often for the patients in the event group (*P*=.02). Indicators for nurse worry that were reported in both groups are specified in Multimedia Appendix 1. In the event group, most reported indicators of worry included “Change in breathing,” “Subjective nurse observation,” and “Patient indicates.” The patient diary results did not differ significantly between the groups (*P*=.35 and *P*=.37). However, patients in the event group seemed to report a (very) poor and a (much) impaired status more often, although these patients filled in the daily diary less frequently. According to the sensitivity analysis, adding the excluded patient group to the control group did not change any of the results, as all *P* values remained within the same levels of significance (ie, *P*<.001, *P*<.05, or *P*>.05).

In the 24 hours before the onset of the included events, the average sensor EWSs had a median value of 3.4 (IQR 2.5-4.0), which was significantly different from the median value of 2.0 (IQR 1.1-2.7) of the average nurse EWSs in this period (*P*=.006) and from the median value of 2.8 (IQR 2.4-3.2) of the average sensor EWSs observed across the total measurement period in the control group (*P*=.04).

**Figure 4:**
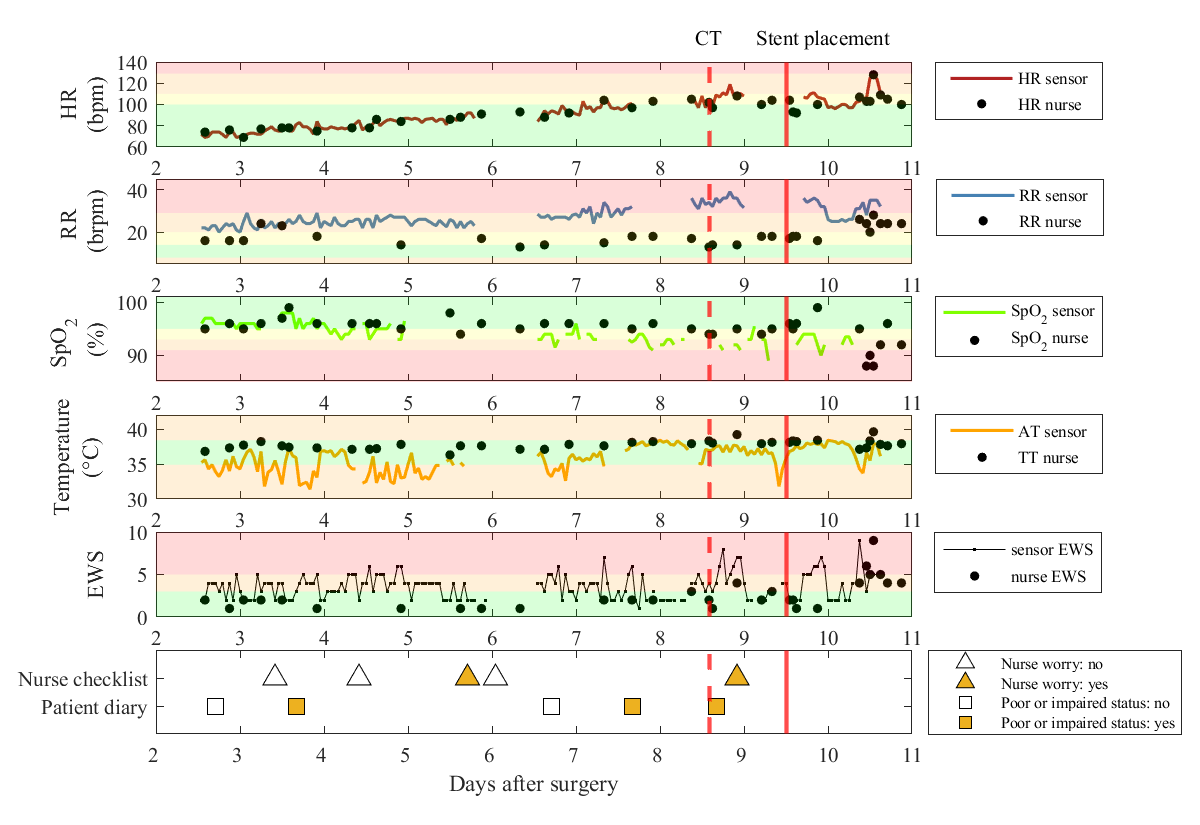
Case example of a patient with anastomotic leakage. AT: axillary temperature; bpm: beats per minute; brpm: breaths per minute; CT: computed tomography; EWS: early warning score; HR: heart rate; nurse EWS: EWS based on manual vital signs measurements; RR: respiratory rate; SpO_2_; blood oxygen saturation; sensor EWS: EWS based on sensor vital signs measurements; TT: tympanic temperature. The sensor vital sign measurements and sensor EWS measurements are calculated using 1-hour windows.

Figure 4 shows a case example of a patient undergoing esophagectomy diagnosed on postoperative day 8 with anastomotic leakage (Clavien-Dindo class IV) using a computed tomography scan. Treatment included the placement of an esophageal stent on day 9 (defined as event onset) and antibiotic therapy, followed by intensive care unit readmission on day 11 due to progressive hemodynamic instability. The sensor recordings contained missing data periods for all the vital parameters. Furthermore, the sensor measurements differed from the nurse measurements, particularly in terms of RR and temperature. The sensor and nurse measurements showed a similar gradually increasing pattern in HR in the days before event diagnosis and treatment. However, the sensor measurements indicated an increasing, although extreme, trend in RR before diagnosis, whereas nurse RR measurements increased only after the stent placement. Correspondingly, the sensor EWSs reached values ≥5 more frequently from day 7, whereas the nurse EWSs reached values of ≥5 only on day 10. In addition to abnormal vital sign measurements, nurse worry as well as impaired or poor self-reported patient status were observed not only in the 24-hour period before stent placement but also earlier in the postoperative trajectory.

**Table 3:** Measurement results in the event and control group.

Result		Event group (n=14), median (IQR)		Control group (n=32), mean (IQR)		*P* value
**Sensor measurements**						
	Measurement period^a^ (h)	186 (92-260)	119 (106-142)		.10	
**Hourly sensor EWSs^b^**						
	Sensor EWS availability (% of the measurement period)	82 (66-98)	85 (68-93)		.94	
	Average sensor EWSs	3.5 (3.1-4.1)	2.8 (2.4-3.2)		.004	
	Sensor EWSs ≥3 (% of available hourly sensor measurements)	62 (44-83)	49 (41-66)		.08	
	Sensor EWSs ≥5 (% of available hourly sensor measurements)	25 (18-40)	8.1 (2.4-17)		<.001	
**Nurse EWSs^c^**						
	Nurse EWSs availability (number of measurements/24 h)	3.4 (2.3-4.2)	2.7 (1.9-3.1)		.02	
	Average nurse EWSs	1.7 (0.9-2.3)	0.9 (0.4-1.3)		.009	
	Nurse EWSs ≥3 (% of available measurements)	22 (7.7-34)	0 (0-12)		<.001	
	Nurse EWSs ≥5 (% of available measurements)	4.3 (0-6.9)	0 (0-0)		<.001	
**Hourly single-parameter recordings**						
	1 parameter out of normal range^d^ (% measurement period)	26 (19-41)	18 (13-25)		.03	
	Multiple parameters out of normal range^d^ (% measurement period)	3.0 (0.0-13)	0 (0-0.8)		<.001	
**Nurse worry checklist**						
	Nurse worry checklist availability (number of checklists filled/24 h)	1.2 (0.8-1.9)	1.2 (0.9-1.6)		.93	
	Nurse worry expressed (% of available nurse worry checklists)	25 (0-33)	0 (0-17)		.02	
**Patient diary**						
	Patient diary availability (number of diary forms filled/24 h)	0.5 (0-1)	0.8 (0-1)		.37	
	Patient expressed (very) poor and (much) impaired status (% of available diary forms)	14 (0-36)	0 (0-33)		.35	

^a^Period of sensor measurements performed during ward stay.

^b^Sensor EWS: early warning score based on sensor vital sign measurements.

^c^Nurse EWS: early warning score based on manual vital sign measurements.

^d^Out-of-normal range conforms to single-parameter alarm criteria.

### Alarm Evaluation

#### Sensor EWS-Based Alarms

**Figure 5:**
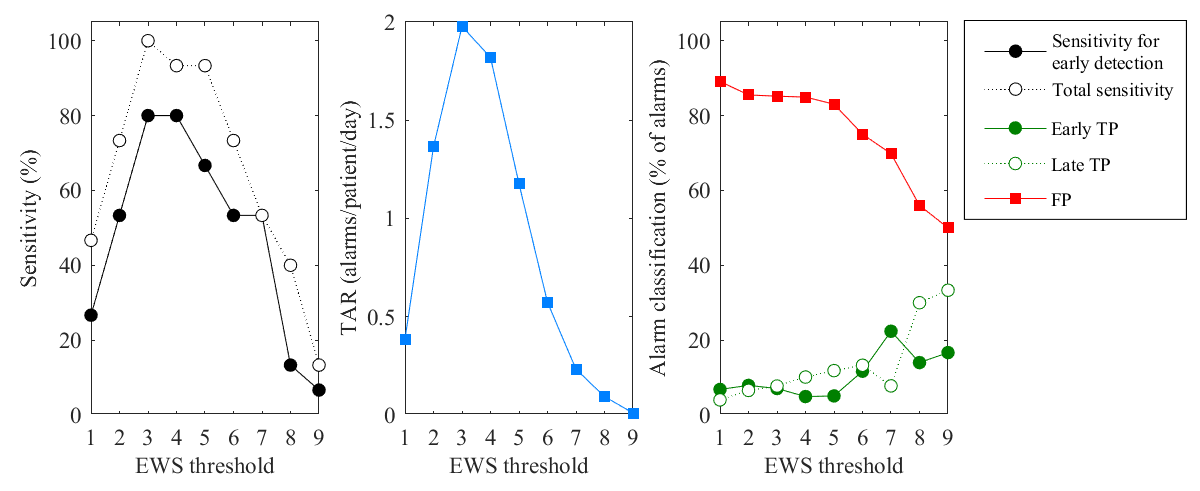
Sensitivity, total alarm rate (TAR), and alarm classification of early warning score based on sensor vital signs measurements (sensor EWS)–based alarms, calculated using 1-hour windows and early warning score (EWS) thresholds of 1 to 9. FP: false-positive; TP: true-positive.

Figure 5 displays the performance metrics of sensor EWSs alarms simulated in 1-hour windows using EWS thresholds of 1 to 9. The sensitivity rates were the highest for an EWS threshold of 3, where alarms were observed 24 hours before the onset of included events in 12 out of 15 events (sensitivity for early detection=80%) and during the treatment period for all events (total sensitivity=100%). TAR followed a similar pattern as the sensitivity rates and reached the highest level for a threshold of 3, resulting in a maximum of 2 alarms per patient per day, of which 85% was classified as FP alarms. An EWS threshold of 5 was associated with a TAR of 1.2 alarms per patient per day, FDR of 83%, sensitivity for early detection of 67%, and total sensitivity of 93%.

**Figure 6:**
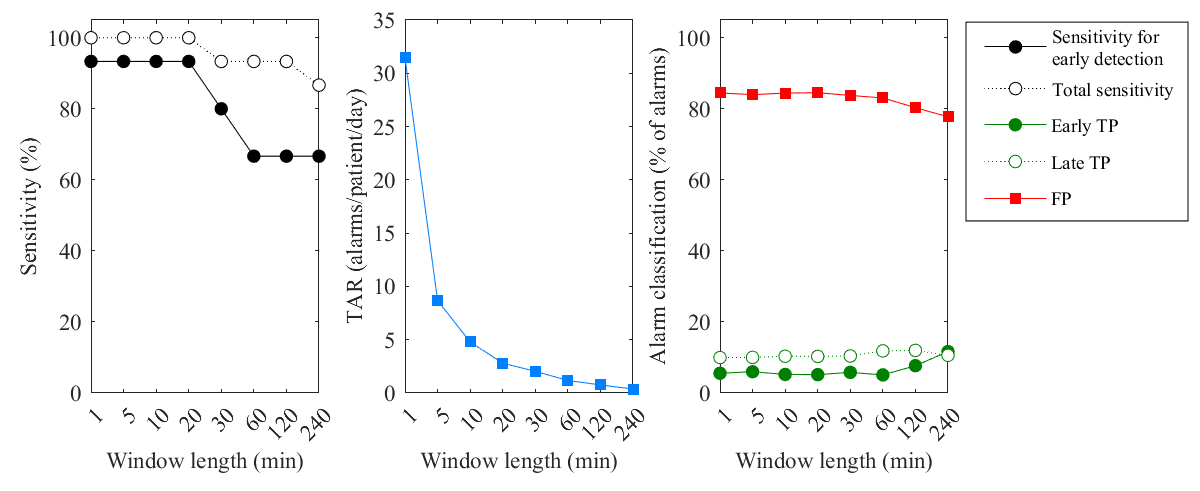
Sensitivity, total alarm rate (TAR), and alarm classification of early warning score based on sensor vital signs measurements (sensor EWS)–based alarms, calculated using 1- to 240-minute windows and an early warning score (EWS) threshold of 5. FP: false-positive; TP: true-positive.

Figure 6 shows the performance of sensor EWSs alarms for window lengths of 1 to 240 minutes, illustrated for an EWS threshold of 5 as an example. The sensitivity rates and FDR decreased for longer windows. TAR strongly declined with increasing window length, ranging from 31 alarms per patient per day for window lengths of 1 minute to 0.8 alarms per patient per day for window lengths of 120 minutes. Similar trends were observed for all other thresholds, although the sensitivity decreased more rapidly with increasing window length for the other thresholds. In addition, FDR increased with the window length for thresholds of <3. The highest TAR levels were observed for a threshold of 3, reaching up to 70 alarms per patient per day for a 1-minute window length.

#### Nurse EWS-Based Alarms

**Figure 7:**
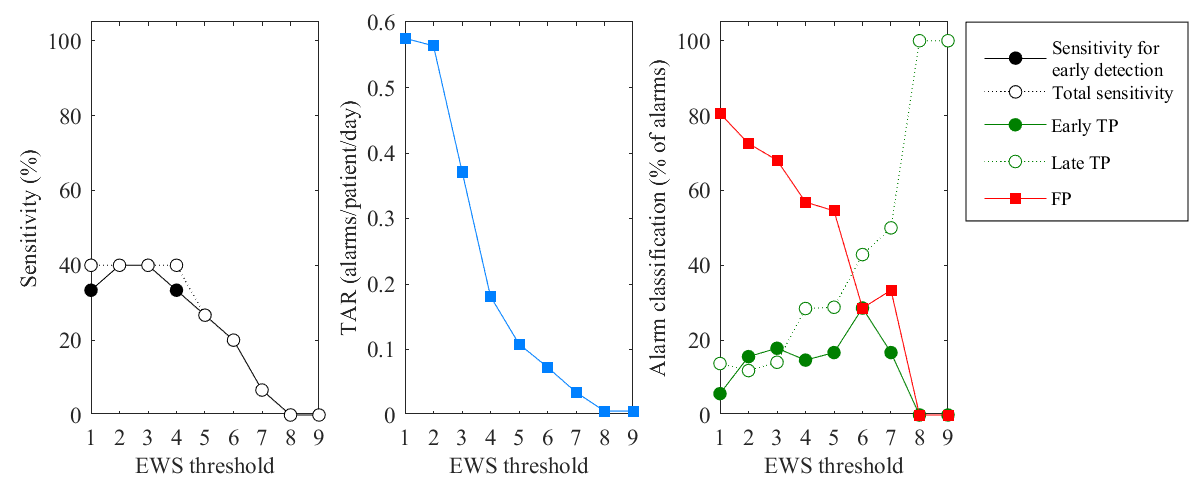
Sensitivity, total alarm rate (TAR), and alarm classification of early warning score based on manual vital signs measurements (nurse EWS)–based alarms, calculated using early warning score (EWS) thresholds of 1 to 9. FP: false-positive; TP: true-positive.

Figure 7 displays the performance metrics of nurse EWS–based alarms. The sensitivity for early detection and total sensitivity were the same for most threshold values, reaching a maximum of 40%. The TAR and FDR were the highest (0.6 alarms/patient/d and 80%, respectively) for a threshold of 1 but decreased for higher threshold values. An EWS threshold of 5 was associated with a sensitivity for early detection and total sensitivity of 27%, TAR of 0.1 alarms per patient per day, and FDR of 54%. Compared with the sensor EWSs, not only the sensitivity rates but also the TAR and FDR of the nurse EWSs were lower for all thresholds >1.

#### Single-Parameter Alarms

**Figure 8:**
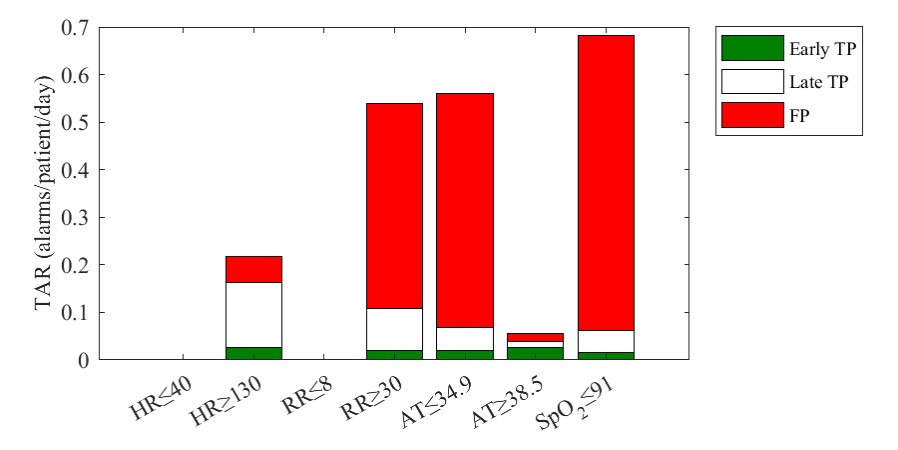
Total alarm rate (TAR) of single-parameter alarms calculated using 1-hour windows. AT: axillary temperature; FP: false-positive; HR: heart rate; RR: respiratory rate; SpO_2_: blood oxygen saturation; TP: true-positive.

Figure 8 shows the TAR and classification of the single-parameter alarms. In total, the single-parameter alarms resulted in a TAR of 2 alarms per patient per day. The FDR of all single-parameter alarms was 83.6%, and 4.7% of the alarms were classified as early TP and 11.7% as late TP. In 13 of the 15 events, at least 1 single-parameter alarm was observed in the 24-hour period before the onset of the event, resulting in a sensitivity for early detection of 87%. Total sensitivity was 100%. Most alarms (>0.5 alarms/patient/d) were observed for low SpO_2_, low AT, and high RR, of which a large part (≥80%) was classified as FP alarms. In contrast, high HR and high AT alarms were associated with lower alarm rates and lower FDR (≤30%). No single-parameter alarms were observed for low HR or low RR.

## Discussion

### Principal Findings

This study investigated the potential usability of high-rate EWSs obtained from wireless vital sign data for early identification of complications in patients on surgical wards and explored the performance of a sensor EWS–based alarm system in comparison with single-parameter alarms and manual EWS measurements by nurses for various alarm settings. The EWSs based on hourly sensor recordings were significantly higher in patients with complications than in patients with an uncomplicated postoperative trajectory. Furthermore, the sensitivity to predict adverse events within 24 hours was higher for sensor EWS–based alarms compared with nurse EWS–based alarms. Therefore, EWSs obtained from sensor measurements might contribute to early awareness or confirmation of patient deterioration in current ward routines. However, high EWSs were often recurrent in patients with and without events and could therefore lead to high false alarm rates when used as real-time alarm systems. Although hourly EWS measurements resulted in fewer alarms compared with single-parameter alarms, the number of false EWS alarms increased when using shorter time windows and varied between alarm thresholds, highlighting the importance of careful selection of alarm settings.

### Comparison With Prior Work

#### Prediction of Complications

Traditionally, vital signs are used to monitor patients after surgery, as vital signs change 4 to 24 hours before adverse events [25]. The additional use of EWSs supports systematic assessment and responses to patient deterioration by nurses [5,26]. Coherently, it can be expected that frequent monitoring of EWSs can contribute to improved and early recognition of complications. This expectation is supported by the current case example and by other studies where abnormal vital signs and corresponding (partial) EWSs were observed more evidently, more often, or earlier in sensor recordings than in nurse measurements [23,27]. Similarly, sensor EWS–based alarms and single-parameter alarms based on hourly sensor measurements provide a higher sensitivity for predicting events within 24 hours compared with nurse EWS–based alarms. RR contributed the most to high sensor EWSs, which agrees with previous studies reporting RR as an important predictor of deterioration [5,7,28]. Furthermore, SpO_2_ abnormalities were often observed, which is in line with the high prevalence of hypoxemic (micro) events found in similar observational studies [27,29].

Compared with hourly sensor EWS–based alarms, we found higher sensitivity rates for single-parameter alarms, although the differences were small for some EWS thresholds. This observation is in line with a study by Rothman et al [30] who reported that only 32% of life-threatening adverse events were preceded by abnormalities in multiple components of the EWS, whereas 27% showed only a single abnormal criterion. In contrast, Rothman et al [30] also reported that 41% of the adverse events were not preceded by abnormal vital signs at all. This raises the question of the relatively high sensitivity of both the sensor EWS–based and single-parameter alarms. Accordingly, it is plausible that abnormal vital sign measurements were not caused by the complication development but were related to normal variations within patients or the postoperative status, as supported by the observation that high sensor EWSs were also present in the control group. Similar findings were reported by Itelman et al [31], who reported that national EWSs based on 5-minute wireless vital sign recordings obtained in patients on general wards provided warnings for deterioration events in 67% of the cases on average 29 hours before detection by caregivers but also led to warnings in 78% of all patients who did not experience deterioration. Similarly, Haahr‐Raunkjaer et al [29] reported that episodes of abnormal vital sign measurements were observed more often during the 24-hour period before events in patients admitted to the ward; however, the duration of abnormalities in continuous recordings did not differ between patients with and without serious adverse events.

Another possible explanation for the low specificity is that sensor-based respiration rates were typically much higher than nurse-based measurements. Whether this reflects the inaccuracy of nurse-based RR measurements [10] or RR overestimation by the sensor remains to be determined. In this context, one should be aware that the overestimation of RR falsely increases EWSs, which reduces the discriminative ability of systems. Together, these findings highlight the risk of event overdetection and highlight the need for further optimization of the combined warning criteria for wireless monitoring.

#### Alarms

The use of automatic alarm systems in monitoring routines can contribute to the early awareness and active responses of care professionals to potential deterioration [32]. However, excessive false alarm rates can lead to alarm fatigue, posing a serious risk to patient safety and a barrier to the adoption of continuous monitoring [33]. Therefore, a balanced ratio between alarm sensitivity, specificity, and alarm rate is crucial, which requires careful selection of alarm thresholds. In current practice, a variety of EWS thresholds are used, depending on the EWS variant and clinical settings. The calculation of the sensor EWSs was based on the MEWS criteria, for which studies recommended thresholds of 3 or 4 for monitoring patients on wards [34,35]. However, the results of this study indicate that the sensor EWSs resulted in the highest false alarm rates for EWS thresholds of 3 and 4. In addition, we found that sensor EWSs ranged between 2 and 4 and rarely dropped to values <2, which also explains the low alarm rates for thresholds of <3. Therefore, using a threshold of 5 may be considered to reduce the alarm burden in sensor-based alarm systems.

Alarm rates based on the hourly sensor EWSs were lower than those based on the hourly single-parameter alarms, which underlines the benefits of a multiple-parameter approach to improving specificity. However, it should be noted that the EWS alarm rates increased rapidly when using shorter time windows. Therefore, it might be appropriate to restrict automatic alarm generation based on EWSs to intervals of 1 hour or even longer in a ward setting and additionally present historical data trends for a more detailed evaluation of patient deterioration during nurse rounds. The use of summary measures for continuously measured vital signs is supported by van Goor et al [36], who reported that mean values calculated over multiple hour time frames can be helpful in supporting the identification of respiratory insufficiency. Although assessing data over longer time windows instead of every few minutes will limit alarm sensitivity, this approach would still be an improvement compared with intermittent nurse observations by allowing more frequent and less-static EWS measurements. Moreover, such an approach might be well accepted, as it protects nurses from alarm overload and promotes the use of the system as a support system without compromising the nurses’ leading role in the decision-making process. For safe and effective implementation, it is crucial that caregivers are trained in interpreting the data and alarms and that high-risk patients requiring continuous surveillance are admitted to a high-care unit with a nurse:patient ratio that allows immediate response in case of acute deterioration [7,37].

#### Sensor Versus Manual Vital Signs Measurements

The use of wireless sensors provides new opportunities for monitoring vital signs in ward settings. By enabling continuous recording, wireless monitoring supports the comprehensive investigation of data abnormalities and trends, reducing the likelihood that signs of deterioration are missed due to intermittent or incomplete nurse measurements [10,27]. In addition, reducing the time needed for routine measurements can reduce nurses’ workloads, leaving more time for patient observation and support. However, remote vital sign monitoring technology is still immature, and standards for the implementation of remote patient monitoring in ward routines are lacking. Furthermore, sensor measurements during patient mobilization still present practical challenges; for example, wireless connection issues and limited patient adherence [27,33] can lead to data loss. In our study, we encountered most measurement issues for SpO_2_, where we found a relatively high rate of missing data related to detachment of the finger probe by patients, loss of Bluetooth connection, and short battery life, as also reported in another study using the same device [29]. Technological improvements can be achieved by developing new sensors that can derive accurate SpO_2_ values from locations on the body that are less prone to (movement) artifacts and sensor dislodgment. Furthermore, adequate methods for correcting missing data periods in postanalysis and real-time alarm systems are desired.

Second, wearable sensor measurements are prone to artifacts caused by motion and poor skin contact, and clinical validation studies have shown variable accuracy of remote systems [38-40]. These sensor inaccuracies could explain the observed low agreement between the sensor and nurse data pairs, where the average bias was particularly large for RR and SpO_2_ as also observed in other studies [23]. However, it must be noted that nurse observations also have variable accuracy, especially for RR [10]. In addition, discrepancies between the sensor and nurse measurements can be expected when measurements are performed using different sensing techniques, sensor locations, and under different circumstances; for example, in this study, sensor measurements recorded AT instead of tympanic temperature and were performed during daily activities, where vital sign measurements can be influenced by physical activity, whereas nurse measurements were performed during rest. Together, these factors underline that sensor measurements and corresponding EWSs are not a one-on-one replacement of measurements performed by nurses, where each approach has its benefits and limitations. These differences highlight the need for appropriate response trigger criteria for continuous monitoring, perhaps requiring different classifications of EWS points or the implementation of alternative detection algorithms.

Finally, when seeking continuous risk prediction methods or fully automatic EWS assessments, it should be considered that currently available wireless monitoring systems are restricted to measuring only a selection of vital parameters. For example, the level of consciousness is implemented in standard EWS or patient monitoring routines [9] but cannot be measured automatically using current sensor systems. Until reliable sensing and preprocessing techniques are available for all relevant parameters, it would be of interest to develop ways to optimally combine sensor and nurse measurements in care routines. Various commercially available systems for wireless or automatic patient monitoring already support or are used for the calculation of complete EWS every few hours [12,41], often after verification of valid measurement values and manual insertion of parameters that cannot yet be measured by the system, such as blood pressure or consciousness. Following this approach, Weenk et al [23] performed a study that included patients on wards, where an EWS was calculated every 30 minutes using 2 different wireless vital sign sensor systems and complementary nurse measurements. Higher EWSs were seen earlier in combined recordings than in nurse measurements alone, suggesting that integration of manual and automatic monitoring could contribute to better continuous patient monitoring and improve early identification of deterioration.

#### Nurse Worry and Patient-Reported Indicators

Subjective evaluation of patient status is an essential element of patient monitoring and clinical decision-making [7,37]. The presence of nurse worry has been described as a predictor of deterioration and is increasingly used as an individual calling criterion or as part of EWS variations [19,42]. The importance of nurse worry was confirmed by this study, where we found that a nurse worry checklist could support the identification of patients with events and may reveal early signs of deterioration, as illustrated by the case example. Accordingly, wireless monitoring should be accompanied by routine nurse evaluations, which could mitigate the risk of overreliance on technology [7]. To ensure consistent collection and valuation of subjective information that cannot be retrieved with sensors, it can be beneficial to embed structured bedside observation scores such as the Dutch Early Nurse Worry Indicator Score [19] in electronic patient files and warning criteria.

In addition to nurse worry, well-being indicators reported by patients or by their relatives provide important signs of deterioration [8], and the presence of patient concerns has been suggested as an additional care escalation criterion [43]. Correspondingly, we found that patient-reported deterioration was often reported as a reason for nurse worry in patients in the event group. However, signs of deterioration are not always recognized or expressed by patients or their relatives [43]. In this study, exploring a patient diary that aimed for the independent and structured assessment of patient indicators, we noticed that patients with complications seemed to report a poor or impaired status more frequently. Despite regular encouragement from researchers to fill in the diary, the responsiveness to it was limited. Interestingly, patients with events seemed less responsive to the diary compared with the control group, which might be a sign of deterioration by itself. Further investigation of patient-reported indicators for use in monitoring routines, perhaps using patient-tailored questionnaires or 2-way communication systems that promote patient response, is recommended.

### Strengths and Limitations

This exploratory study included 2 surgical populations with a relatively high risk of developing postoperative complications and, therefore, may benefit from improved monitoring. The heterogeneity of our study population and the variety of included events allowed the exploration of the predictive value of continuous EWSs for different algorithm settings. However, the total number of included patients and events eligible for analysis were limited, and it should be noted that clinical deterioration may present differently across other settings. Therefore, validation of this study’s results in larger cohorts and other specific or general populations is needed once remote monitoring systems are mature and implemented more widely. Similarly, verification of the performance of high-rate EWSs for monitoring systems using other sensors or different parameter sets is desired. Finally, prospective studies assessing the clinical effects of using high-rate EWSs are needed.

The criteria used for the simulation of the sensor EWSs were based on clinical standards to allow comparison with current practice; therefore, the optimization of alarm criteria was limited to exploring different cutoff thresholds and sample frequencies. Furthermore, the EWSs were obtained using average vital sign values, whereas the use of other summary measures can result in different EWSs [44]. Because the results of this study revealed that sensor EWS–based alarms could lead to overdetection and high false alarm rates, further investigation of the best-suited preprocessing methods as well as alternative alarm criteria or methods is highly recommended; for example, it might be beneficial to personalize the allocation of EWS points by adapting normal ranges based on previous recordings of individuals or patient groups [24] or to correct for the influence of physical activity using embedded motion sensors. Moreover, because patterns of deterioration depend on the underlying cause [2] and patient characteristics, it could be useful to implement algorithms tailored to detect specific complications or patient groups [45]. Finally, trend-based metrics, machine learning techniques, or dynamic models may provide better performance for identifying abnormal patterns in continuous vital sign data compared with traditional EWSs, and event prediction may be improved further by integrating additional patient information extracted from the electronic patient record [16,28,45-51].

Although many studies have focused on the prediction of life-threatening events such as cardiac arrest, this study focused on all minor and major complications that required additional care in a ward setting. Patients with Clavien-Dindo class I complications that, by definition, did not require pharmacological or surgical treatment were excluded from the analysis. The sensitivity analysis indicated that the inclusion of these patients in the control group did not affect the results, indicating that the predictive value of vital signs or EWSs is limited for these events. Following the concept that early warning systems should alert only when action is required, this study used the onset of therapeutic actions as a surrogate marker of complication onset. However, the timing of therapeutic onset depends highly on the diagnostic definitions and is prone to delays in diagnostic or therapeutic activities. Furthermore, delays in clinical reporting hamper accurate retrospective determination of therapy onset. Therefore, prospective clinical trials are needed to investigate the effects of using a high-rate sensor-based EWSs as a diagnostic tool or alarm system on time to treatment and the corresponding patient outcomes.

### Conclusions

Hourly EWSs obtained using wireless vital sign sensors resulted in higher sensitivity for predicting postoperative complications compared with the currently used 8-hour, nurse-based EWSs. Therefore, sensor EWSs may contribute to the improved detection of clinical deterioration in patients on surgical wards. In addition, using the EWS as an active alarm trigger results in fewer alarms compared with single-parameter alarms in sensor measurements. However, false alarm rates can increase rapidly when calculating EWSs in short time spans, risking alarm fatigue and overdetection. To prevent alarm overload in a ward setting, we suggest restricting EWS-based alarm generation to windows of at least 1 hour or longer. In addition, investigation of alternative care escalation criteria for wireless monitoring is warranted because automatic vital sign recordings differ from nurse measurements and are currently only available for a selected set of vital parameters. Finally, it is recommended to embed a structured collection of subjective nurse and patient information into monitoring routines, as this might provide complementary signs of deterioration. Future clinical studies are needed to evaluate the clinical effects of using the EWSs and the corresponding alarm system for continuous monitoring of patients on wards.

## Appendix

Multimedia Appendix 1 Nurse worry indicators.

Multimedia Appendix 2 Bland-Altman analysis.

## Abbreviations

AT: axillary temperature
bpm: beats per minute
brpm: breaths per minute
EWS: early warning score
FDR: false discovery rate
FP: false-positive
GI: gastrointestinal
HR: heart rate
MEWS: Modified Early Warning Score
nurse EWS: early warning score based on manual vital signs measurements
RR: respiratory rate
sensor EWS: early warning score based on sensor vital signs measurements
SpO_2_: blood oxygen saturation
TAR: total alarm rate
TP: true-positive

## Data Availability

The deidentified vital sign measurements that were analyzed during this study are available from the corresponding author upon reasonable request.
