# Supplementary material for: Early Warning Scores to Support Continuous Wireless Vital Sign Monitoring for Complication Prediction in Patients on Surgical Wards: Retrospective Observational Study
Source: JMIR Perioper Med. 2023 Aug 30;6:e44483. doi: 10.2196/44483 (PMC10500362; doi:10.2196/44483)
Supplement: Multimedia Appendix 1 [file periop_v6i1e44483_app1.pdf]

Nurse worry indicators

Table S1. Nurse worry indicators

Nurse worry indicator	Underlying signs and symptoms
Change in breathing	Noisy breathing<br>Short of breath<br>Unable to speak full sentences<br>Use of accessory muscles
Change in circulation	Color changes: pile /grey<br>Sweaty / clammy<br>Coldness<br>Impaired perfusion<br>Oedema
Temperature	Rigors
Change in mentation	Apathic / lethargic<br>Confused
Agitation	Restless<br>Anxious
Pain	New pain<br>Increasing/persistent pain
Unexpected trajectory	No progress<br>Abdominal distension / nausea / vomiting<br>Bleeding<br>Dizzyness<br>Fainting<br>Falls
Patient indicates	Not feeling well<br>A feeling of impending doom
Family indicates	Change in behavior<br>Change in attitude<br>Doesn’t look good<br>Look in the eyes
Subjective nurse observation	Change in behavior<br>Change in attitude<br>Doesn’t look good<br>Look in the eyes
Other	Any other reason for nurse worry

The nurse worry indicators were adapted from the Dutch-Early-Nurse-Worry-Indicator-Score (DENWIS) described by Douw et al. [19] and used for reporting reasons for nurse worry in the ‘Nurse worry checklist’.

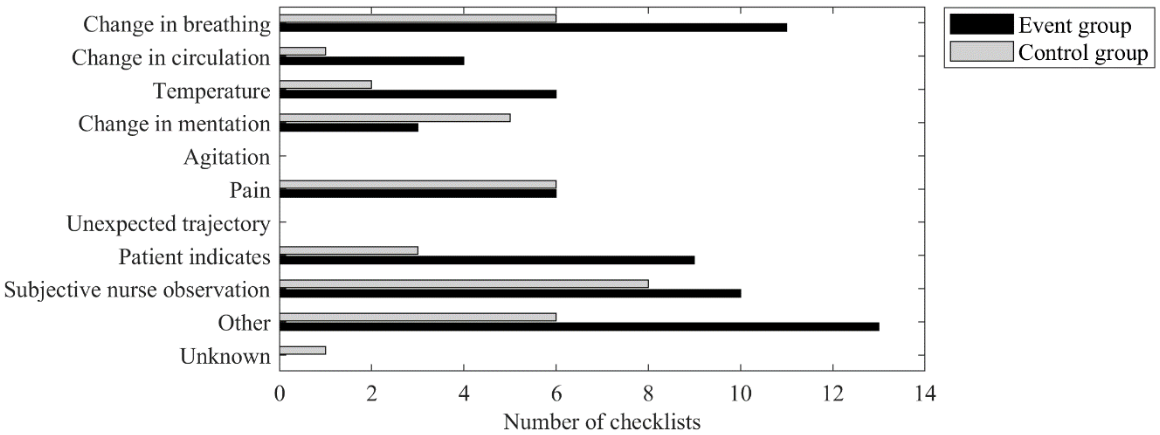

Figure S1. Prevalence of indicators for nurse worry (specified in Table S1) as reported in the nurse worry checklists in the patients with complications (event group) and patients with uncomplicated postoperative trajectory (control group).
