# Supplementary material for: Early Warning Scores to Support Continuous Wireless Vital Sign Monitoring for Complication Prediction in Patients on Surgical Wards: Retrospective Observational Study
Source: JMIR Perioper Med. 2023 Aug 30;6:e44483. doi: 10.2196/44483 (PMC10500362; doi:10.2196/44483)
Supplement: Multimedia Appendix 2 [file periop_v6i1e44483_app2.pdf]

Bland-Altman analysis

Figure S1 displays the Bland-Altman plot, showing the difference between nurse and sensor measurements compared to the corresponding average value of the measurements, for each available data pair (i.e. pair of nurse measurement and the corresponding average value of sensor measurements in the prior 5-min window) in the total study population (n=60). The color intensity of the hexagons is proportional to the number of data pairs found in the range outlined by the hexagon. For example, for HR, we observed many data pairs with an average value (i.e. the average of the nurse and sensor data pair) of 80 bpm and a bias (i.e. the difference between the nurse and sensor data pair) of -3 bpm as indicated by the yellow hexagon, whereas only a few data pairs were found with an average HR value of 140 and a bias of -3 bpm. Since the average bias (black dashed line) was negative for HR (-3 bpm) and RR (-9.4 brpm), it can be concluded that the sensor measurements were higher than nurse measurements, on average. Oppositely, the nurse measurements were on average higher than sensor measurements for the SpO_2 and temperature, as the bias was positive. For each of the parameters, we observed relatively large 95% limits of agreement (red dashed lines), indicating large variations in mutual differences between sensor and nurse measurements. The largest deviations were particularly seen for higher measurements for HR and RR and for the lower range measurements for SpO_2 and temperature, as observed in the right lower and left upper corners of the graphs respectively.

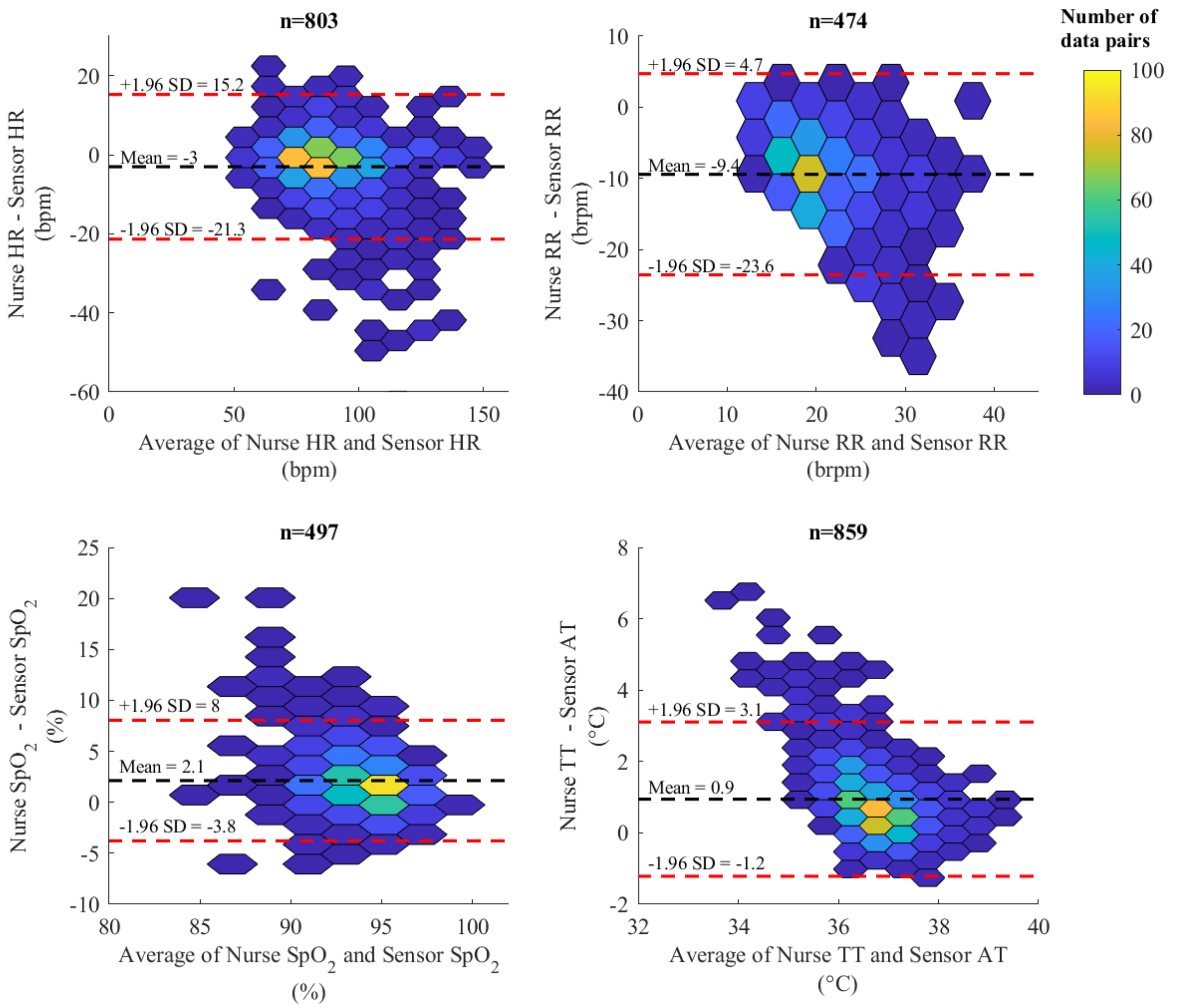

Figure S1. Bland-Altman plot of agreement between nurse and sensor measurements. AT: axillary temperature; HR: heart rate, n: total number of data pairs; RR: respiratory rate; SD: standard deviation; SpO_2: blood oxygen saturation; TT: tympanic temperature.
